# Proximity Labeling Reveals How Lrp2 Interacts with the Endocytic Machine

**DOI:** 10.1021/acs.jproteome.5c01053

**Published:** 2026-04-20

**Authors:** Tian H. Shen, Andrew Beenken, Hediye Erdjument-Bromage, Ora A. Weisz, Aryan Ghotra, Jared S. Kushner, Rachel E. Sturley, Atlas Kahn, Leora Kronenberg, Gabriel Rahmani, Kivanc Nesanir, Frances A. High, Patricia K. Donahoe, Jonathan Barasch, Thomas A. Neubert

**Affiliations:** † Department of Medicine, 5798 Vagelos College of Physicians and Surgeons, Columbia University, New York, New York 10032, United States; ‡ Department of Neuroscience, 5894 New York University Grossman School of Medicine, New York, New York 10016, United States; § Department of Medicine, University of Pittsburgh School of Medicine, Pittsburgh, Pennsylvania 15261, United States; ∥ Massachusetts General Hospital, Harvard Medical School, Boston, Massachusetts 02114, United States

**Keywords:** Megalin, APEX2, proximity biotinylation, proximity labeling, Lrp2

## Abstract

LRP2 (Megalin or low-density lipoprotein-related receptor 2), together with Cubilin and Amnionless, is responsible for binding and internalizing a wide range of nutrients and toxins from the kidney’s glomerular filtrate by endocytosis. Accordingly, Lrp2 deletion or mutation results in the loss of these ligands into the urine. Yet Lrp2 is essential not only for receptor-mediated but also for fluid-phase endocytosis, implicating a broader role beyond ligand binding. To identify the linkage between Lrp2 and endocytosis, we engineered Lrp2-APEX2-expressing mice and performed biotinylation in vivo to label Lrp2’s cytoplasmic partners. We demonstrated the specificity and sensitivity of this technique by mass spectrometric identification of biotinylated proteins from kidney lysate and immunostaining kidney sections. We identified critical endocytic regulators interacting with Lrp2, but also many proteins functionally associated with endocytosis that are not already known to interact with Lrp2. These data suggest that Lrp2 plays a central role in organizing apical membranes through PDZ domain proteins and engages with regulators and molecular motors during endocytosis. These interactions are abolished in the absence of Lrp2.

## Graphical abstract

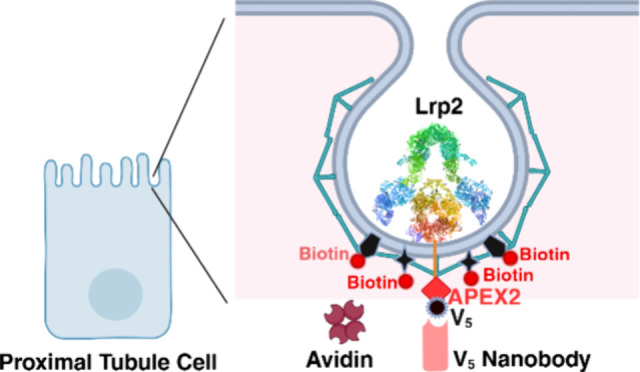

## Introduction

Microvilli (brush border) and networks of "subapical endosomes’ are distinct features of the luminal surface of epithelial cells in many parts of the body, including the kidney’s proximal tubules.^1^ The microvilli expand the cell’s contact with the surrounding fluid and enhance nutrient uptake and waste discharge via transmembrane carriers and transporters,^2–6^ while subapical endosomes capture macromolecular ligands. Megalin, or LRP2, is an endocytic receptor critical for the capture of numerous ligands. It is located in the apical membrane of proximal tubular cells, including microvilli, but most prominently in subapical endocytic vesicles.^7,8^ It is unknown how Lrp2 shuttles from microvilli into endosomes at the base of the microvilli and subsequently into an apical recycling compartment carrying ligands.

The critical role of Lrp2 in the capture of macromolecules has been demonstrated in knockout models of mice,^9–11^ zebrafish,^12^ and in Lrp2-expressing OK cells in vitro.^13^ Not only do these models demonstrate loss of ligand capture, but remarkably, the endocytic system of coated pits and dense apical tubules that are apparent in wild-type mice is absent in Lrp2 knockout mice.^9,10^ In fact, apical endocytosis of both receptor-bound ligands and fluid-phase markers is inhibited in knockout mice^9,11^ and zebrafish.^12^ It remains a puzzle how Lrp2 contributes not only to the endocytosis of its cognate ligands but also to organizing the apical endocytic machine of the kidney’s proximal tubule, responsible for multiple forms of endocytosis.

To address these important questions about Lrp2 function, we combined proximity labeling, affinity purification, and mass spectrometry to identify the partners of Lrp2 that mediate endocytosis. By molecular genetic engineering of enzymes, proximity labeling allows large-scale interrogation of proteins, particularly in the intracellular cytoplasm, with exceptional precision.^14^ This is mainly because reactive radicals generated from the enzymatic reaction during labeling are not membrane-permeable and decay rapidly, resulting in effective labeling of proteins at a distance of less than 20 nm.^14,15^ The labeled proteins can be identified by mass spectrometry^16^ after affinity enrichment. Using an improved APEX2 enzyme^17,18^ and recombineering,^19^ we have generated Lrp2-APEX2 mice, and labeled proximal tubule proteins in the proximity of the cytoplasmic tail of Lrp2, and identified these proteins by mass spectrometry. We found dozens of known regulators of apical endocytosis as well as many new candidates. Our investigation demonstrates the power of proximity labeling proteomics to open a rich field of inquiry regarding the functional organization of apical membranes, where endocytosis is the main critical function.

## Experimental Section

### Generation of *Lrp2* ^*APEX2/+*^ Mouse

Mouse *Lrp2* spans 160 kbp. We used a BAC clone (bMQ120-m21, Source Biosciences)^20^ spanning the 3′ *Lrp2* gene. “*Linker-APEX2-V5-frt-neo-frt”* cassette was generated by directional cloning of Linker-APEX2-V5 in the PL451 plasmid at *Kpn*I and EcoR I sites with APEX2 cDNA, previously described.^17–19^ Using recombineering, the Lrp2 *translational STOP (TAG)* was replaced with the “*LinkerAPEX2-V5-frt-neo-frt”* cassette. The modified BAC clone was confirmed by junctional PCR with forward primer (5′–3′): GAACATAAAGCCATGTCATCC, reverse primer (5′–3′): GTTAGCGCTGTGAGCCAGTTC for 5′ junction, and forward primer (5′–3′): GATTCGCAGCGCATCGCCTTC, reverse primer (5′–3′): GATATACACAGTACCTCATG for 3′ junction. The modified region was retrieved from a targeting plasmid. The resulting plasmid was confirmed by junctional PCR with forward primer (5′–3′): GATTAAGTTGGGTAACGCCAG, reverse primer (5′–3′): CTGCTTGCCTACCTCCATGAC for 5′ junction, with forward primer (5′–3′): GATGCCCACGTCTCGTCTGAAG, reverse primer (5′–3′): ATTCGCCAATGACAAGACGCTG for 3′ junction. The targeting plasmid was verified by restriction with NotI and PacI, and finally, the entire “*Linker-APEX2-V5-frt-neo-frt”* cassette, as well as the 5′ and 3′ regions of the insertion site, were completely sequenced to verify the in-frame fusion of APEX2 with Lrp2, connected by a linker and terminated by the V5 tag. We used standard homologous recombination in mouse ES cells (KV1 ES line, 129B6N hybrid ES line, Columbia University Herbert Irving Comprehensive Cancer Center: Genetically Modified Mouse Models Shared Resource). The correctly targeted ES cells were identified by long-range PCR using forward primer (5′–3′) CCACTACCTCCCTCTTCTTCAG, and reverse primer (5′–3′) GTTAGCGCTGTGAGCCAGTTC for 5′ junction, and forward primer (5′–3′) GATTCGCAGCGCATCGCCTTC and reverse primer (5′–3′) GCTGGGTCTGGAAACTGGAAC for 3′ junction. *Lrp2* ^*APEX2/+*^ mice were generated by the blastocyst injection of targeted ES cells. The in-frame fusion of APEX2 to the C-terminus of Lrp2 permits APEX expression in the cytoplasm of cells wherever Lrp2 is expressed.

The Neo cassette was removed by crossing with *Actin-Flpe* mice (JAXMICE), and the resulting *Lrp2* ^*APEX2/+*^ mice were maintained as heterozygotes. The homozygotes were fertile, and no abnormalities were observed. The mice were genotyped using primers (5′–3′): GTTCGACTCCAGGCTACACTG (forward), CTCAGCGATGAAGCCTCTGAG (reverse 1), and ACCTCCTGACAACATCAGTGC (reverse 2), resulting in 191 and 304 bp fragments for the mutant and wild-type alleles, respectively.

### Perfusion of *Lrp2* ^*APEX2/+*^ Mouse Kidney Ex Vivo

Kidneys were harvested from mice according to IACUC protocols and methods were approved and established at Columbia University by Liu et al.^18,21^ Mice were inoculated with 5000 U/kg of heparin sodium (25021-400-30, Sagent Pharmaceuticals) *i.p.* 5 min before euthanasia to improve renal perfusion post-mortem. The kidney was immersed in physiological saline (NaCl 141.8 mmol/L, KCl 4.7 mmol/L, MgSO4 1.7 mmol/L, ethylenediaminetetraacetic acid 0.5 mmol/L, CaCl_2_ 2 mmol/L, HEPES 10 mmol/L, KH_2_PO_4_ 1.2 mmol/L, glucose 5 mmol/L; Sigma-Aldrich) and the renal artery exposed and cannulated. Perfusion was carried out at a constant pressure of 60 mmHg using a peristaltic pump (PS200, Living Systems Instrumentation) in a blood vessel perfusion chamber (CH1, Living Systems Instrumentation) at a slowed rate of ∼2 mL/min. The kidney was first perfused with permeant biotin-phenol (0.5 mM, 5 mL; Iris LS-3500) in the EDTA/HEPES buffer described above for 25–40 min. Subsequently, freshly prepared H_2_O_2_ (0.3%) in PBS was perfused for 5 min, and then ice-cold quenching buffer (sodium ascorbate 10 mM, sodium azide (10 mM), in 25 min PBS). While 5 min perfusion is longer than used by other investigators to label cells directly in culture,^17,22,23^ the longer time ensured sufficient peroxide perfused the afferent arteriole of the nephron, was filtered across the glomerulus, and into the lumen of the nephron, contacting the proximal tubule. This was confirmed by perfusing the renal artery with bromophenol blue (1 mg/mL; B-6131, Sigma-Aldrich), which demonstrated intact perfusion and urine flow to the bladder. Also noted that while only 1 min of labeling with H_2_O_2_ was used in cardiac studies in mice with transgenic expression of APEX2 fused to calcium channels, the perfusion rate was nearly 4-fold faster in the mouse heart than in the kidney (∼2–8 vs ∼1–2 mL/min).^18^ Hence, while biotin phenoxyl radicals are thought to last less than 1 ms in the cytosol and in this way covalently modify neighboring proteins within 20 nm, by extending the perfusion of H_2_O_2_, we might have labeled proteins beyond the nanodomain of the channel. However, the most abundant proteins detected after affinity purification are likely in closer proximity to LRP2, including Lrp2 itself and many proteins known to be closely associated. Further, the C-terminus of LRP2 is adjacent to the inner leaflet of the membrane and thus probably limits the extent of labeling. Quality control for labeling included staining with fluorescent avidin to detect sites of biotinylation and GO enrichment analysis showing brush border proteins concordant with the subcellular localization of mature LRP2.

### Isolation of *Lrp2* ^*APEX‑V* **5** */+*^ Biotinylated Proteins

The perfused kidney was homogenized in 125 mM TEAB, 75 mM NaCl, with phosphatase and protease inhibitor tablets (900 μL) using a sterile disposable pestle fitting into an Eppendorf tube. SDS (final 1% SDS) was added, mixed well, incubated on ice for 15 min, and then the lysate was sonicated (10 × 10 s/each on ice with 10 s pauses between each pulse. The lysate was centrifuged (10,000*g* at 4 °C for 15 min), and the supernatant was adjusted to 300 μg of protein/200 μL of TEAB buffer (no SDS, added 20 μL of 0.2 M DTT), rotated for 30 min, and then treated with freshly prepared iodoacetamide (0.2 M, 60 μL) at 23 °C for an additional 30 min. Subsequently, streptavidin magnetic beads (150 μL) were added with rotation for 90 min. The beads were washed with TEAB without SDS (0.5 mL), then with 1 M KCl (0.5 mL), and then 5 times with 0.1 M TEAB (0.5 mL). New tubes were used after each wash. The washed beads were frozen in 0.1 M TEAB buffer (30 μL, −80 °C). In some cases, beads were analyzed by Western blot using streptavidin-HRP (1:5000), comparing input (10.5 mg), washes (10.5 mg), and 10% of beads.

### Biotinylation of Kidney Sections of Lrp2^APEX2‑V5/+^ and Wild-Type Mice

Kidneys were sliced using an Alto stainless steel coronal brain matrix (Roboz, Cat. No. SA-2150), with Feather double-edge carbon steel blades (Ted Pella, Inc., Cat. No. 21-9). The sections (0.5 mm) were washed with PBS and then incubated in permeant biotin-phenol (0.5 mM) in PBS (23 °C; 1 h), and then 0.3% H_2_O_2_ for 2 min, followed by ice-cold quenching buffer. Sections were then fixed in 4% PFA overnight and embedded in OCT for immunofluorescence.

### Protein Identification and Quantitation by Liquid Chromatography–Mass Spectrometry (LC–MS/MS) Using Label-Free Data-Independent Acquisition (DIA)

The relative abundance of proteins captured by streptavidin magnetic bead extractions of Lrp2^APEX2^-mediated biotinylated kidney lysates was determined by label-free relative quantitative proteomics (MaxLFQ) using data-independent acquisition (MaxDIA).^24^ Streptavidin magnetic bead-bound proteins were subjected to reduction and alkylation of disulfide bonds, and after 3 washes with digest buffer, they were incubated at 37 °C and digested overnight with 500 ng mass spectrometry-grade trypsin (Trypsin Gold, Promega, Madison, WI, USA) in 50 mM NH_4_HCO_3_ digest buffer. After acidification with 10% formic acid to a final 1% formic acid, peptides were extracted with 5% formic acid/50% acetonitrile (v/v) and concentrated to a small droplet using vacuum centrifugation. Desalting of peptides was performed using hand-packed SPE Empore C18 Extraction Disks as described.^25^ Desalted peptides were again concentrated and reconstituted in 10 μL of 0.1% formic acid in water. Aliquots of the peptides were analyzed by nano-LC-MS/MS using an Easy nLC 1000 equipped with a self-packed 75 μm × 20 cm reverse phase column (ReproSil-Pur C18, 3 μm, Dr. Maisch GmbH, Germany) coupled online to a Q Exactive HF Orbitrap mass spectrometer via a Nanospray Flex source (all instruments were from Thermo Fisher Scientific, Waltham, MA, USA). Analytical column temperature was maintained at 50 °C by a column oven (Sonation GmbH, Germany). Peptides were eluted with a 3%–40% acetonitrile gradient over 110 min at a flow rate of 250 nL/min. The mass spectrometer was operated in data-independent acquisition mode. MS survey scans were acquired in profile mode at a resolution of 120,000 (at *m*/*z* 200) over a scan range of 300–1650 *m*/*z*. Following the survey scans, 30 groups of precursors were selected for fragmentation with sliding isolation windows to include peptide *m*/*z* values ranging from 364 to 1370 Th. The default maximum charge state was set to 4, and resolution was set to 30,000. In MS/MS, the fixed first mass was set to 200 Da. The normalized collision energy (NCE) /stepped NCE was 25.5, 27, and 30. The maximum injection time for the survey scan was 60 ms, and for MS/MS, it was set to auto. The ion target value for both scan modes was set to 3 × 10^6^. Data were analyzed by MaxQuant software (version 2.5.1.0), termed the MaxDIA analysis type. To identify peptides, we used mouse in silico-generated spectral libraries that were created from the UniProt mouse protein sequence database (downloaded on 06/18/2020; 21,989 entries) from the Max Planck Institute of Biochemistry data-sharing drive (MPIB Datashare https://Datashare.biochem.mpg.de/s/qe1IqcKbz2j2Ruf?path=%2FDiscoveryLibraries). To determine the relative abundance of a protein across a set of samples, we used its label-free quantitation (MaxLFQ) intensity calculated by MaxDIA,^24,26^ which normalizes the protein intensity based on peptide intensities across samples.

### Statistical Analysis of Mass Spectrometry Data

We compared gene-level log2-transformed intensity values between the two experimental groups (APEX and no APEX, *n* = 4 mice per group) using a Welch’s *t-*test. For each gene, log2 intensities from replicates (Intensity_APEX1–APEX4 and Intensity_no APEX1–no APEX4) were used directly without additional transformation. Genes with insufficient replicates (more than two missing values per group) or zero variance in either group were excluded from testing. For each protein, we calculated the mean intensity per group and the log2 fold change (difference between group means on the log2 scale) as an effect size. Raw *p*-values were adjusted for multiple testing using the Benjamini–Hochberg false discovery rate (FDR) to generate *q*-values. Results shown in Supplementary Table 1 include *p*-values, *q*-values, mean intensities, and log2 fold changes for downstream analysis.

### OptiPrep Gradient and Fractionation

One mouse kidney was minced with a razor blade and then Dounce homogenized with 10 strokes of loose followed by tight pestles. Homogenization buffer (7 mL) consisted of sucrose (0.235 M), Tris-HCl pH 7.5 (20 mM, Corning, Cat No. 46-030-CM), PMSF (50 mg/mL, Research Products International, Cat. No. P20270), aprotinin (1 μg/mL, Sigma, Cat. No. A1153), pepstatin (1 μg/mL, Roche Diagnostics, Cat No. 10253286001) and leupeptin (5 μg/mL, Roche Diagnostics, Cat. No. 11017101001). The homogenate was centrifuged (1000*g* for 10 min at 4 °C; Beckman Allegra 6KR centrifuge; Beckman Coulter Inc., NJ, USA) and the pellet was collected, rehomogenized, and recentrifuged. Supernates were combined and recentrifuged again, and the final supernate precipitated (10,000*g* for 10 min at 4°) using a Sorvall RC-5B centrifuge (Thermo Fisher Scientific, MA, USA). The pellet was resuspended in 350 μL of homogenization buffer and layered atop an OptiPrep (Sigma, Cat. No. D1556) step gradient of 6.9%, 12%, 16%, and 20% OptiPrep in homogenization buffer with protease inhibitors. The gradient was spun at 13,200 × *g* for 60 min in a Beckman L7 Ultracentrifuge and then fractionated into 40 fractions using the Model 152 Piston Gradient Fractionator (Biocomp Instruments, New Brunswick, Canada). Membrane fractions were pelleted using a S120AT3-0033 Rotor; 100,000xg, and protein concentrations were determined by BCA (Thermo Fisher Scientific, Cat. No. 23227).

To test the capture of a known ligand of Lrp2, *Lrp2* ^*APEX2‑V5/+*^mice were inoculated i.p. with myoglobin (1 mg; Millipore Sigma # M0630) 10 min prior to kidney fractionation.

### Isolation of *Lrp2* ^*APEX2/+*^ Labeled Endosomes

OptiPrep fractions containing Lrp2^+^ membranes were pooled, and *LRP2* ^*APEX‑V5*^ endosomes were isolated with V5-nanobodies (80 μL of V5 nanobeads; ChromoTek V5-Trap Magnetic Particles M-270, Proteintech Cat. No. v5td) from pooled OptiPrep fractions (1.8 mL) mixed for 90 min. The beads were washed three times with cold PBS, eluted at 4 °C with V5 peptide (20 μL of 0.7 mM ChromoTek V5-peptide, Proteintech Cat. No. v5p; 15 min), and then with 2× SDS sample buffer (95 °C for 5 min). Eluted beads were removed with a magnetic stand. We used antibodies anti-V5 (ThermoFisher Scientific, Cat. No. R960, 1:5,000), anti-Lrp2 (Abcam, Cat. No. ab76969; 1:3000), anti-Albumin (Bethyl, Cat. No. A90-134A 1:1000), and antimyoglobin (Santa Cruz, Cat. No. sc-393020; 1:200).

### Urine NGAL

This biomarker of injury was measured in the urine of male and female wild-type mice and LRP2-APEX-V5^**+**^ mice. Urine was spun (12,000*g* × 10 min), and aliquots containing equal amounts of urine creatinine (Abcam, Cat. No. ab65340) were analyzed by immunoblot using antimouse NGAL (Bioporto, Cat. No. ABS 043-10).

## Results

The cytoplasmic tail of Lrp2 contains domains or motifs that recruit the endocytic machinery to carry out ligand uptake and transport inside the cell, but also to recycle back to the plasma membrane or to lysosomes for the degradation of aged receptors.^27^ Consequently, many different intracellular binding partners of Lrp2 are implicated. To identify these interactors, we generated an in-frame C-terminal fusion of Lrp2 and APEX2 cDNA, flanked by a linker for proper folding of the APEX2 enzyme and a V5 tag for immunodetection of the fusion protein (Figure 1). The Lrp2^APEX2‑V5/+^ mice were healthy and fertile. In addition, the mice had normal Lrp2 function. For example, the proximal tubule cells of Lrp2^APEX2‑V5/+^ mice function like those in wild-type mice, and capture both fluorescent dextran (Figure 2), a fluid phase marker, as well as albumin and myoglobin protein ligands (see below). In addition, the Lrp2^APEX2‑V5/+^ mouse did not demonstrate evidence of glomerular or tubular injury: neither proteinuria nor urinary NGAL (a marker of tubular injury) was elevated, nor was there histological evidence of injury (Supplemental Figure 1). In contrast to Lrp2^APEX2‑V5/+^, Lrp2-deleted mice, Lrp2^5′Cre/f^ mice cannot capture dextran, and they transcribe NGAL RNA and produce high levels of urinary NGAL protein (Figure 3).

**Figure 1:**
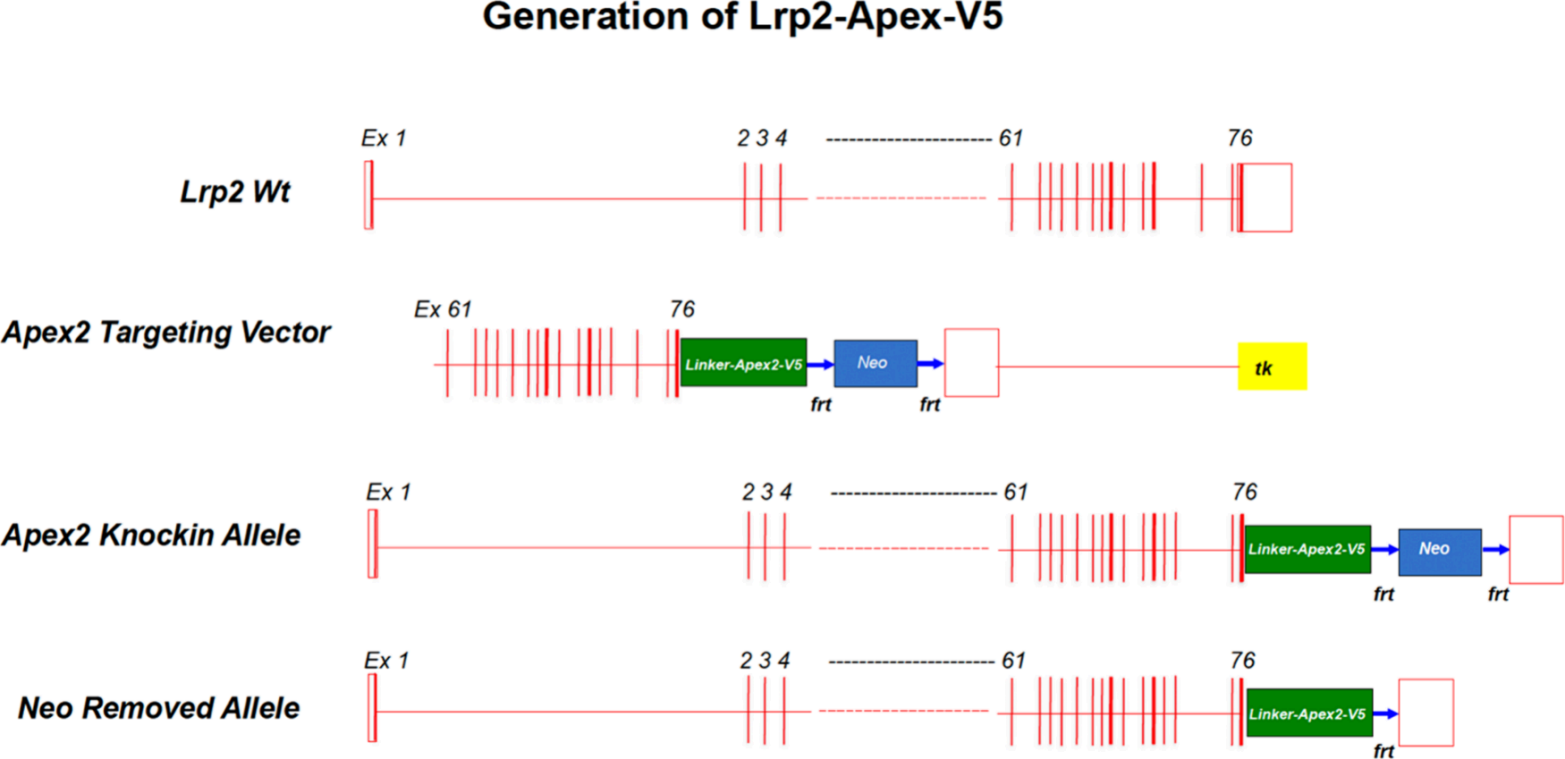
Generation of *Lrp2* ^*APEX2‑V5/+*^ mouse. The mouse *Lrp2* gene spans 160 kbp. We used a BAC clone containing the 3′ *Lrp2* gene to generate *Lrp2* ^*APEX2/+*^. Using recombineering, we replaced the *translational STOP (TAG)* with the “*LinkerAPEX2-V5-frt-neo-frt”* cassette and sequenced the entire cassette as well as the 5′ and 3′ sequences of the insertion sites to verify the in-frame fusion of APEX2 with Lrp2 connected by a linker and ending with a V5 tag. *Lrp2* ^*APEX‑V52/+*^ mice were generated by standard homologous recombination in mouse ES cells and blastocyst injection into targeted ES cells. The Neo cassette was removed by mating with *Actin-Flpe* mice, and the resulting *Lrp2* ^*APEX2/+*^ mice were maintained as heterozygotes. The *Linker-APEX2-V5-frt-neo-frt* cassette was not drawn to scale within the *Lrp2* gene.

**Figure 2:**
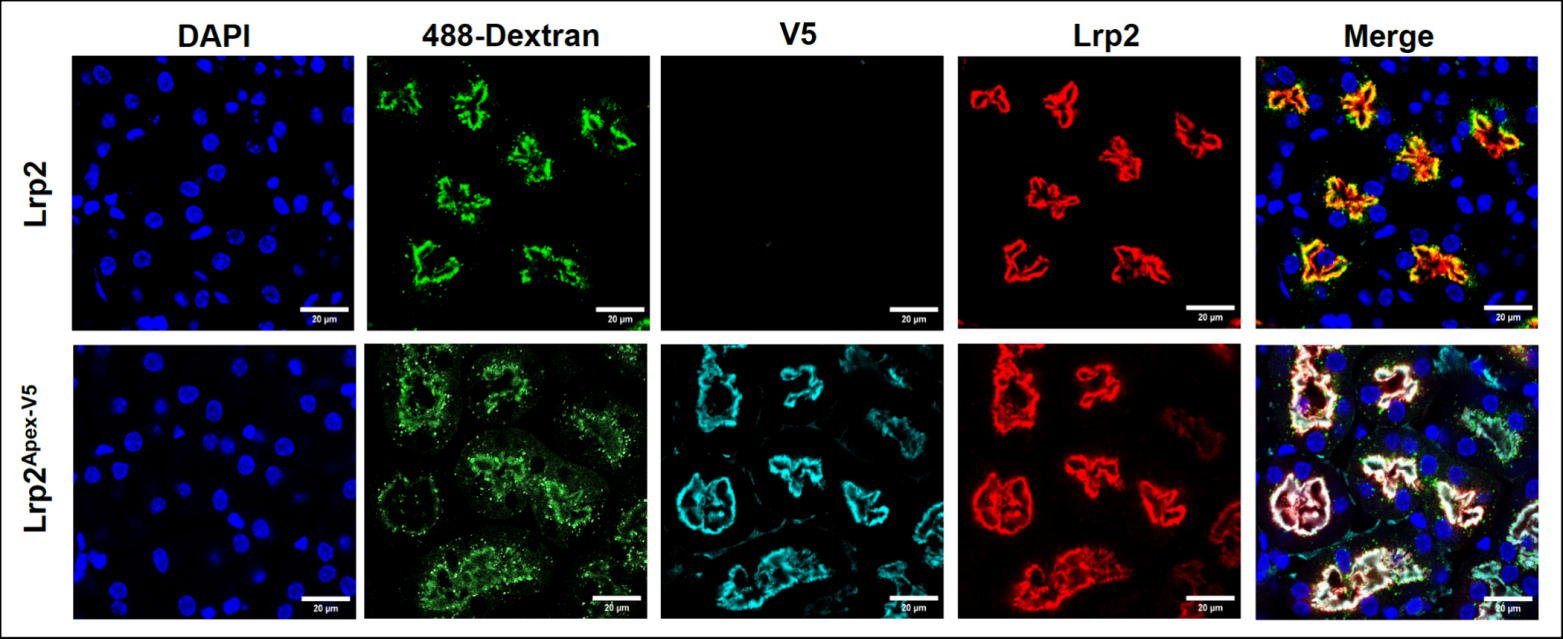
Endocytic function of *Lrp2* ^*APEX2‑V5/+*^ mouse is preserved. 488-Dextran (250 mg) was captured by the proximal tubular cells in both wild-type (Lrp2) and Lrp2^APEX2‑V5/+^ mice. Note that the expression patterns of V5 and Lrp2 proteins are identical and overlap with those of captured dextran.

**Figure 3:**
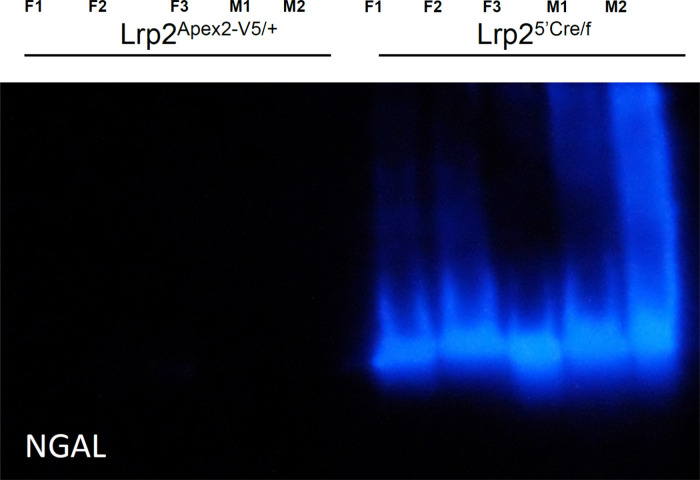
NGAL protein immunoblot shows that urine NGAL—a biomarker of kidney injury—is not elevated in Lrp2^APEX2‑‑V5/+^ mice. Comparison with the deletion of Lrp2 in the proximal tubule of the kidney. Equal amounts of urine creatinine were loaded onto each lane. F, female M, male.

The synthesis of the Lrp2^APEX2‑V5/+^ fusion protein was detected in the kidney extracts: the C-terminal most, “V5” tryptic peptide of the fusion protein “Activity Enhanced APEX2” (Seq ID: QOE77465.1) – V5 peptide LSELGFADAGKPIPNPLLGLDST was detected by DDA as well as by DIA acquisition methods as an experimental mass in MS1. Fragmentation spectra (MS/MS) confirmed the analysis (Supplemental Figure 2). In contrast, similar preparations from wild-type mice failed to produce evidence of this peptide in DIA analyses.

To test whether the engineered fusion construct was functional, we purified apical membranes from Lrp2^APEX2/+^ mice and used the membranes as a source of the APEX2 enzyme; addition of the APEX substrate biotin-phenol created an in vitro biotinylation assay. We confirmed APEX2 activity by immunodetecting biotinylated proteins pulled down by streptavidin magnetic beads. H_2_O_2_, the known catalyst, was required for the reaction (Figure 4A).

**Figure 4:**
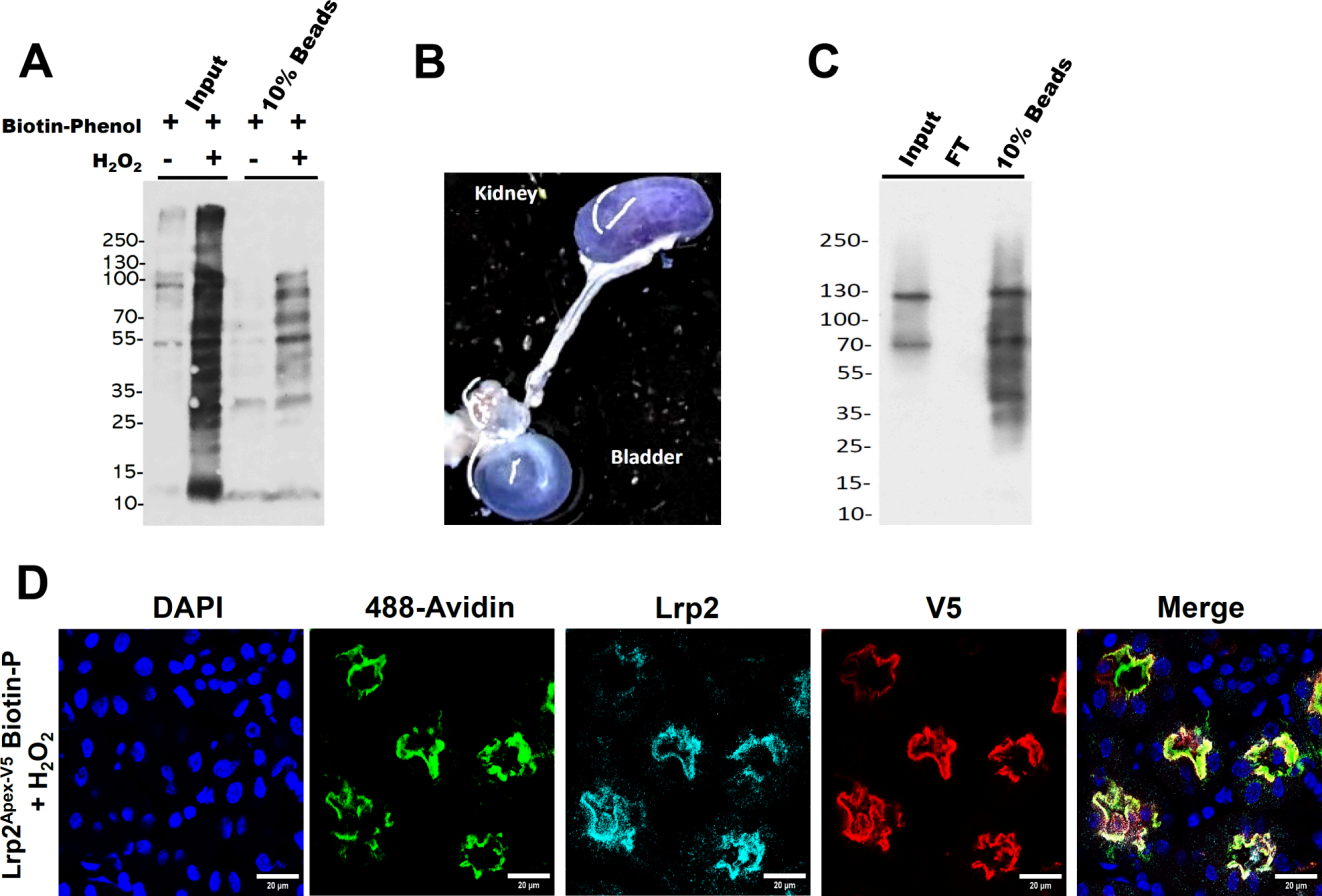
Proximal tubules are biotinylated in vitro and in vivo. (A) *Lrp2* ^*APEX‑V52/+*^ proximal tubule membrane fractions were biotinylated in vitro in the presence of H_2_O_2_, and biotinylated proteins were precipitated with streptavidin magnetic beads. The samples were analyzed by Western blot using HRP-conjugated streptavidin (1:5000). (B) Renal artery was cannulated and perfused with bromophenol blue (1 mg/mL), producing blue urine filling the bladder. (C) *Lrp2* ^*APEX2‑V5/+*^ kidney was perfused with biotin phenol and H_2_O_2_, and biotinylated proteins were precipitated with streptavidin magnetic beads. The samples were analyzed by Western blotting using HRP-conjugated streptavidin (1:5000). (D) Expression of the LRP2^APEX2‑V5^ fusion protein in vivo. Note that Lrp2 and V5 immunoreactivity colocalize with biotinylation, which is detected by apical staining with 488-Avidin in the proximal tubule.

The data demonstrate that we can use subcellular fractions to perform biotinylation and identify biotinylated proteins by mass spectrometry,^28,29^ but we reasoned that live kidney biotinylation identifies endogenous protein–protein proximity. First, we demonstrated that we could perfuse the renal artery, filter a blue dye into the urinary space, and collect the perfusate in the connected bladder (Figure 4B). Then we activated biotinylation by sequential perfusion with biotin-phenol and H_2_O_2_ and isolated biotinylated proteins from a lysate of the perfused kidney (Figure 4C). In tissue sections, the biotinylated proteins, depicted by the avidin binding sites, colocalized with Lrp2 and the V5 tag of APEX2-V5 (Figure 4D).

To demonstrate the specificity of the labeling, we cut 0.5 mm kidney sections of Lrp2^APEX2‑V5/+^ mouse kidneys and performed biotinylation directly on the sections. The biotinylation was restricted to the apical pole of the cell where most of Lrp2 is located and was strictly correlated with the presence of Lrp2 and APEX2-V5 (Figure 5). H_2_O_2_ was required for the biotinylation reaction.

**Figure 5:**
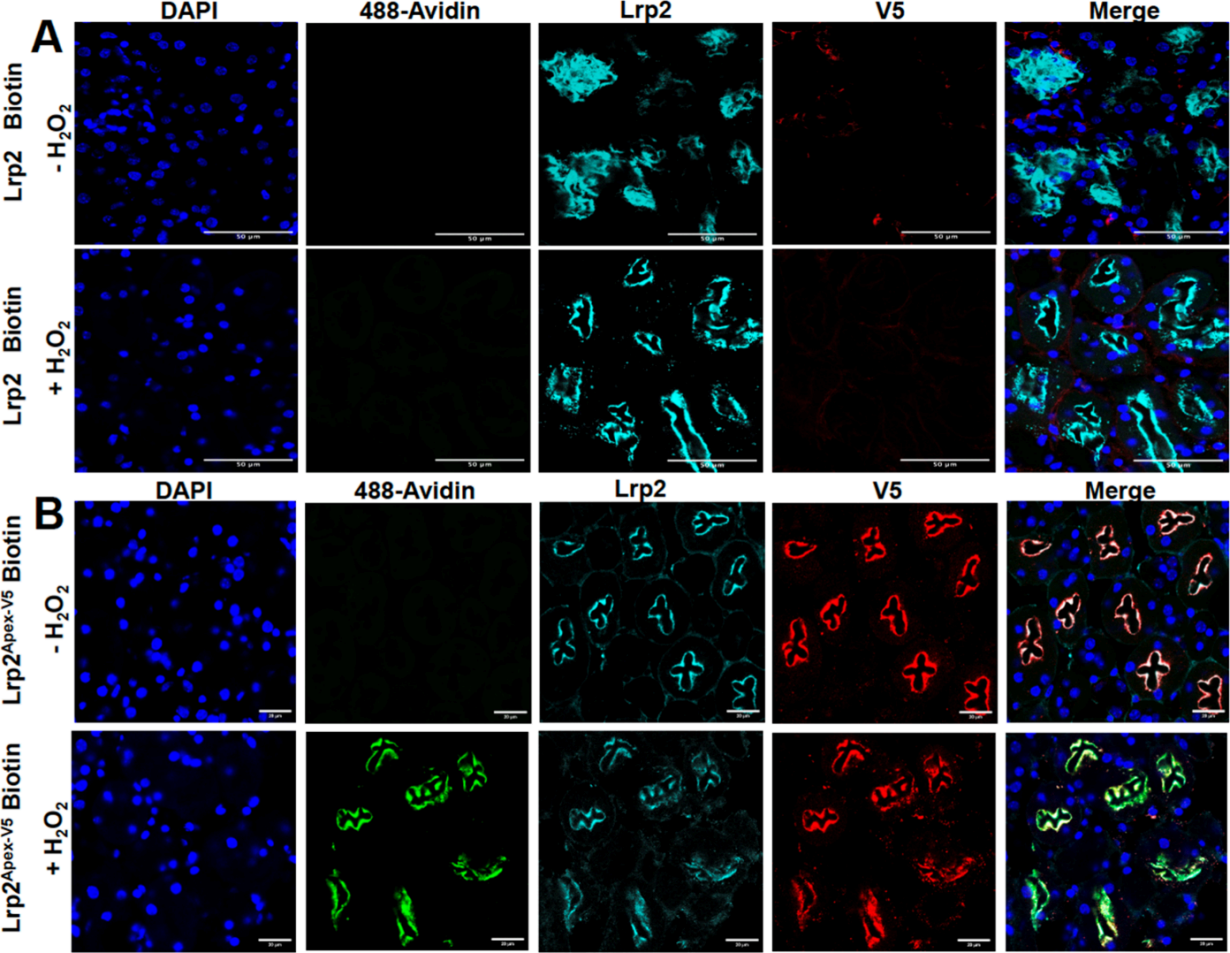
Specificity of LRP2^APEX2‑V5/+^. Fresh, unfixed kidney thick sections (0.5 mm) were treated with biotin-phenol. (A) Wild-type kidney sections. Note the absence of both the V5 c-terminal tag and biotinylation (streptavidin staining). (B) Lrp2^APEX2‑V5/+^ kidney sections depict both the V5 c-terminal tag and biotinylation (streptavidin staining). Biotinylation exclusively colocalizes with Lrp2 and V5 immunoreactivity in the apical domain of the proximal tubule. Biotinylation is dependent on H_2_O_2_.

The specificity of the biotinylation reaction meant that Lrp2-associated proteins could be identified by isolating biotinylated proteins from the perfused kidneys of Lrp2^APEX2‑V5/+^ mice using avidin beads, followed by on-bead trypsin digestion, rather than by attempts at elution, which have yielded inconsistent results. The resulting peptides were analyzed by mass spectrometry for protein identification and quantification. We used wild-type mouse kidneys processed the same way as the Lrp2^APEX2‑V5/+^ mice to control for nonspecific binding to the avidin magnetic beads and to identify endogenous biotinylated proteins (known to be predominantly acetyl-CoA carboxylase and biotin carboxylase)^30^ (Supplemental Table 1 and Supplemental Figure 3).

An extended list of proteins was highly biotin-enriched from Lrp2^APEX2‑V5/+^ mice compared to Lrp2 mice lacking APEX. The list included Dab2 (47-fold increase over background labeling; *p*-value 2.2 × 10^–5^), Amn (42-fold increase; *p* = 1.25 × 10^–2^) and Slc34a1 (48-fold increase; *p* = 2.92 × 10^–4^) which are well-known interactors of Lrp2, but also many other proteins including transporters, small GTPase Rab proteins, and cytoskeleton proteins (Supplemental Table 1). We verified the expression of a few targets, including apical membrane proteins, by immunostaining and found that they colocalized with the Lrp2-V5 tag and with Lrp2-directed biotinylation, marked by 488-Avidin at the initiation point of the proximal tubule in Bowman’s Capsule (Figure 6).

**Figure 6:**
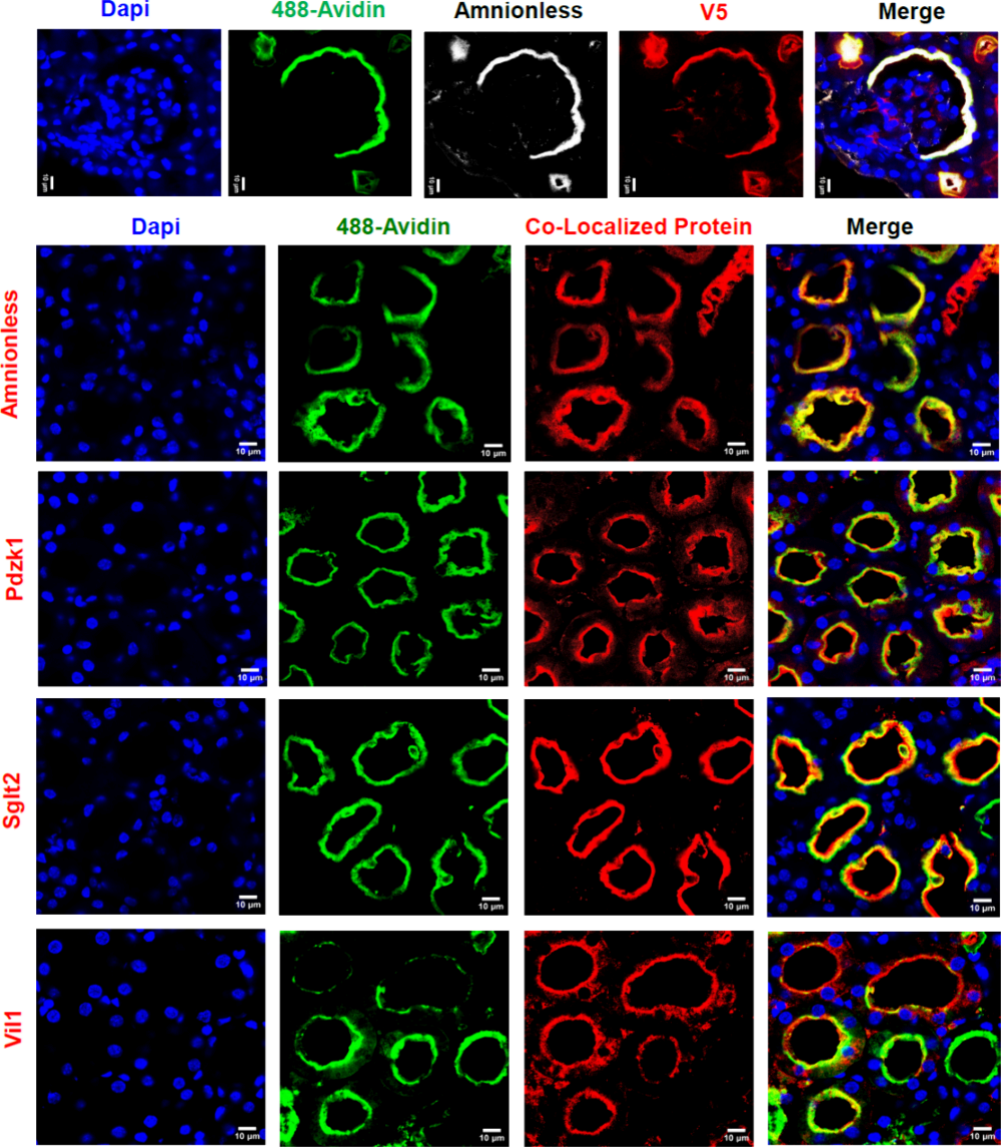
Localization of LRP2^APEX2‑V5/+^ biotinylated proteins. Lrp2 C-terminal-dependent biotinylation in vivo identified cytoplasmic neighbors. Colocalization of biotinylation (detected by 488-avidin) with target proteins and with the V5 tag begins at the tubular pole of the glomerulus at the initiation of the proximal tubule epithelia (top panel). Colocalized protein refers to staining for the proteins named on the left side of the lower panels.

An analysis of the avidin-captured proteins using STRING Gene Ontology^31^ indicates enrichment in brush border (cellular component) and binding to actin filaments/cellular projections (molecular function) (Supplemental Table 2 and Supplemental Figure 4), revealing prominent paths taken by Lrp2 yet to be explored.

Finally, we prepared kidney homogenates from Lrp2^APEX2‑V5/+^ mice and isolated Lrp2^+^-expressing endosomes using anti-V5 nanobodies. The pulldowns concentrated not only on Lrp2 itself but also on the associated protein receptor, Cubilin, and their ligands, albumin and myoglobin (Figure 7). Lrp2^APEX2‑V5/+^ also immunoprecipitated with and biotinylated DAB2, which links Lrp2 to clathrin in endosomes, and immunoprecipitated with and biotinylated Eea1 and Rab7, which define early and late endosomal compartments (Supplemental Figure 5 and Supplemental Table 1). These data support the utility of Lrp2^APEX2‑V5/+^ mice to identify proteins located in Lrp2^+^ endosomes, functionally associated with Lrp2 in the kidney’s proximal tubule.

**Figure 7:**
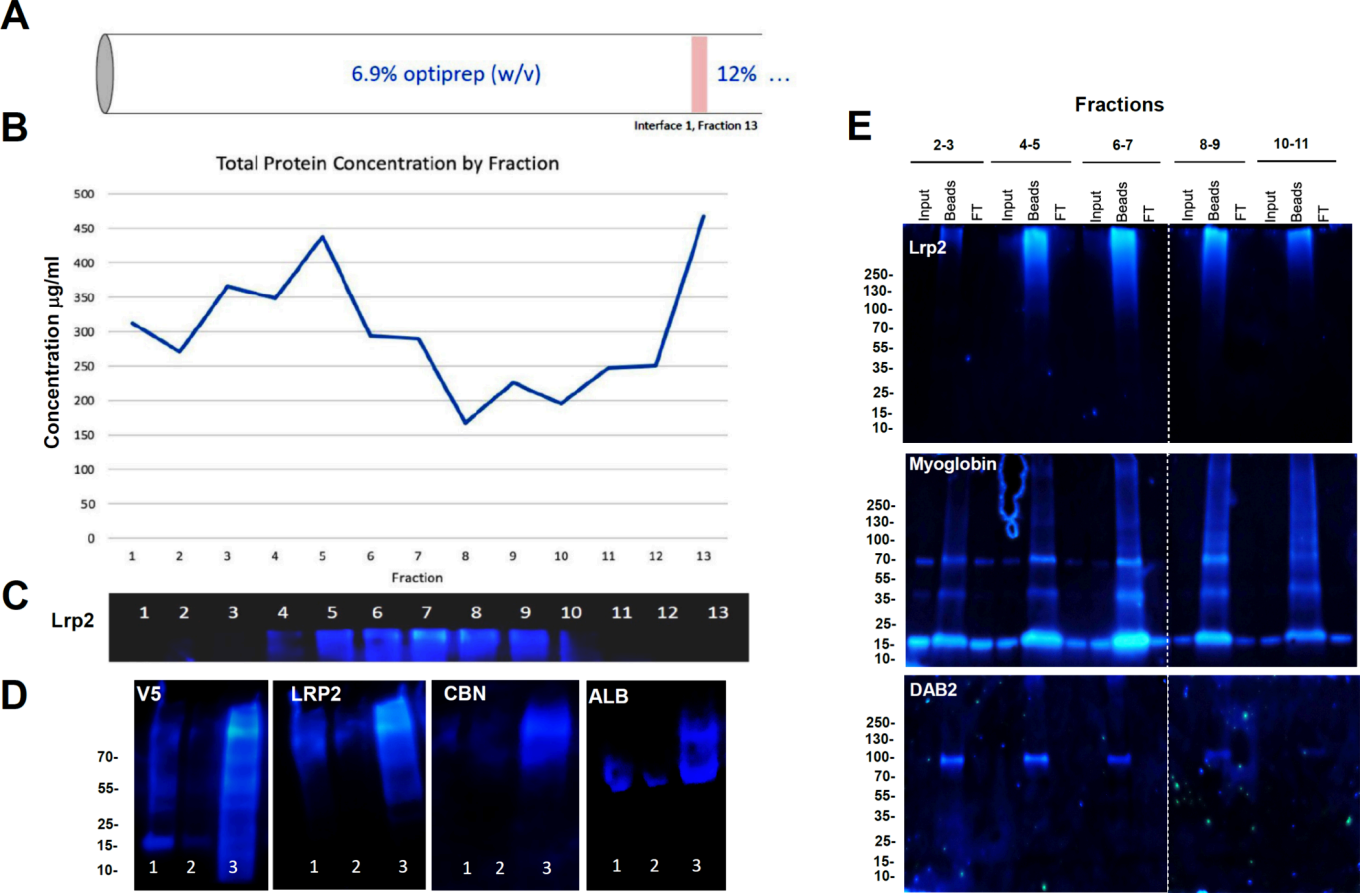
Immuno-isolation of endosomes containing c-terminal Lrp2^APEX‑V5^. (A) Kidney membrane fractions were prepared on an OptiPrep step gradient (top two steps are shown). (B) Bradford protein assay of each gradient fraction. (C) Lrp2 immunoblot of gradient fractions (with equal protein loading). Lrp2 is concentrated in lanes 6–8. (D) Western blot showing immuno-isolation of Lrp2^APEX‑V5^ endosomes from OptiPrep fractions 6–8 using anti-V5 nanobody beads: Lane 1 = OptiPrep fractions; Lane 2 = nanobody bead flow-through; Lane 3 = Capture of V5^+^, Lrp2^+^, Cubn^+^, Alb^+^ endosomes. (E) Western blot showing introduction of myoglobin prior to kidney fractionation on OptiPrep step gradients and anti-V5 nanobody bead pulldowns from different pooled OptiPrep fractions (2−3, 4−5, 6−7, 8−9, 10−11). Myoglobin is found in Lrp2^+^ endosomes along with the Lrp2 endocytic adaptor protein, DAB2.

## Discussion

Lrp2 (low-density lipoprotein-related receptor 2, Megalin) provides a major defense against the loss of protein in the urine. Loss-of-function of Lrp2 leads to proteinuria and a syndromic form of chronic kidney disease, called Donnai–Barrow syndrome (DBS).^32–34^ Additionally, GWAS studies link LRP2 variants to population-wide chronic kidney disease.^35–39^ The broad impact may be attributable to Lrp2’s capacity, unlike any other receptor in the human body, to capture numerous ligands from the urine, many of which are nutrients, and several of which are kidney toxins.^34^ Consistently, the knockout of Lrp2 leads to a near-complete loss of apical endocytosis, implicating Lrp2 as a critical regulator of membrane flux.^40,41^ Beenken et al.^42^ solved the structure of Lrp2, which demonstrated extensive conformational changes, including the repositioning of the intracellular C-terminal domain, according to extracellular or endosomal pH.^42,43^

To shed light on protein partners of Lrp2 at the plasma membrane and in endosomes, we demonstrated for the first time the feasibility of using mass spectrometry to identify the Lrp2 protein interactome both in vivo and ex vivo. We fused APEX in-frame with the cytoplasmic tail of Lrp2 and identified endogenous proteins using APEX-mediated biotinylation while maintaining wild-type gene dosage and expression of Lrp2. This achievement makes it possible to probe and map protein interactions at a scale and to study the reabsorption kinetics of LRP2 ligands under various perturbations of medical relevance, such as volume depletion or acute kidney injury.

### Lrp2 C-Terminal APEX2-Mediated Labeling Is Sensitive and Specific

There are several lines of evidence indicating that our approach is valid. We show that biotinylation activity depends on the presence of both APEX2 and H_2_O_2_; and the biotinylated proteins colocalized with Lrp2 and APEX2, revealed by the V5 tag and by avidin binding sites. We identify biotinylated proteins enriched by avidin pulldowns from Lrp2^APEX‑V5^-expressing kidneys compared with parallel pulldowns from kidneys expressing wild-type Lrp2 and hence screen out the few endogenously biotinylated proteins.^30^ The approach yielded several known proteins interacting with Lrp2, described as follows.

In a yeast two-hybrid screen, Dab2, disabled protein 2, was found to interact with the C-terminal ΨXNPXY (Ψ is any hydrophobic amino acid) of Lrp2 through its phosphotyrosine interaction domain. The interaction was confirmed by coimmunoprecipitation in vitro and in vivo from renal cell lysates.^44^ The localization of Dab2 and Lrp2 in the proximal tubules is mutually dependent. Specifically, Lrp2 remains in microvilli and is restricted from endosomes if Dab2 is lost. Lrp2 is required for the intracellular distribution of Dab2 in the coated pits, while no such localization was observed in Lrp2-deleted renal cells.^45^

Amn, or Amnionless, is a transmembrane protein that is required for Cubilin (lacking a transmembrane domain) cell surface localization and function. Amn and Cubilin (together CUBAM) colocalize in the apical membrane of proximal tubules in the human kidney, and copurify from a human kidney lysate on a gel filtration column.^46^ Loss of Amn causes redistribution of Cubilin from the apical cell surface, but has little effect on the localization of Lrp2.^47^ It has been proposed that Cubilin and Lrp2 function together as major scavenger receptors responsible for capture of nearly all ligands from the kidney filtrate,^48^ and in fact are linked through Cubilin’s extracellular cub domains.^49^ Identification of biotinylated Cubilin is unexpected, as the receptor has no intracellular domain but presumably represents residual association with Lrp2 or with the CUBAM transmembrane subunit Amnionless during isolation of biotinylated proteins. Consistent with this, fold change recovery is ∼3× greater for biotinylated Amn compared with biotinylated Cubn (3:1) even though the complex has a 1:3 Amn:Cubn stoichiometry.^50^

The absence in our list of proteins expected to interact with or modify Lrp2 at the cell surface demonstrates the topological specificity of our approach. The absence of biotinylated RAP (*Lrpap1*) confirms the selectivity of this approach for cytoplasmic rather than luminal partners. Quantitative proteomics finds that there are roughly twice as many copies of RAP per cell as Lrp2.^51^ RAP and Lrp2 interact with a low nM affinity at neutral pH, yet RAP was not biotinylated. Similarly, extensive glycosylation of Lrp2 occurs in its extracellular domain.^52^ During Golgi transit, the 42 predicted N-glycosylation sites on Lrp2 are trimmed, elongated, and modified by a collection of glycosyltransferases and glycosidases, yet no glycosylation enzymes were identified in our screen. The absence of this group of proteins may be a limitation of the assay, but the bulk of these enzymes is located at the luminal face of Lrp2 and has extremely short cytoplasmic termini and hence, may escape proximity ligation. Finally, despite the presence of albumin and myoglobin in the lumen of Lrp2^+^ V5-captured endosomes, we did not identify specific biotinylation in these vacuolar ligands of Lrp2.

A few proteins found in basolateral membranes of the proximal tubule at steady state were biotinylated by Lrp2^APEX^, including aquaporin 1 (*Aqp1*), OAT1 (*Slc22a6*), OCT1 (*Slc22a1*), OCT2 (*Slc22a2*), and a single Na/K-ATPase subunit (*Atp1b1*). However, the FDR for proteins within the GO term “basal part of the cell” (2.80 × 10^–5^) was significantly weaker than that for the “apical part of the cell” (1.73 × 10^–17^), implying that these proteins may have been labeled while trafficking with Lrp2 along the early biosynthetic pathway.

### Lrp2 C-Terminal-APEX2 Identifies Endocytic and Biosynthetic Machinery of Proximal Tubule

As predicted for a membrane receptor that transits rapidly through the apical endocytic pathway, endomembranes, endocytic vesicles, early endosomes, and recycling endosomes are significant pathways found by gene ontology analysis (Supplemental Table 2, FDR 10^–12^–10^–8^). Lrp2 encounters and biotinylates numerous cytoplasmic proteins involved in endocytic membrane trafficking, including its own adaptor protein disabled-2 (*Dab2*), adaptor protein AP-2 complex subunits (*Ap2a2, Ap2a1, Ap2b1, Ap2m1*), clathrin heavy (*Cltc*), epidermal growth factor receptor pathway substrate 15 (*Eps15*), Rab proteins (*Rab5a, Rab5c, Rab7a, Rab10, Rab11, Rab14,* among others), early endosomal antigen 1 (*EEA1*), sorting nexins (*Snx1, Snx3, Snx6*), syntaxin 7 (*Stx7*; involved in late endosome to lysosome transport), and lysosomal-associated protein 1 (*Lamp1*), and vacuolar H^+^-ATPase subunits (*Atp6v1b2, Atp6v0d1, Atp6v1e1, Atp6v1c1, Atp6v1g1, Atp6v1a, and Atp6v0a4*) were also identified. Additionally, several GTPase-activating proteins stimulate the hydrolytic activity of small GTPases that function along the endocytic pathway, including Arfgap1 (*Smap1*), Rabgap1, Tbc1d1, and Rho GTPase-activating proteins Arhgap1 and Arhgap18 (*Arhgap1, Arhgap18*).

The inclusion of several proteins associated with biosynthetic transport, including the endoplasmic reticulum coatomer II subunit sec23a (*Sec23a*) and the α subunit of the COP1 coatomer complex (*Copa*), which are involved in ER-Golgi transport, as well as subunits of the *trans*-Golgi localized adaptor protein 1 complex (*AP1b1, Ap1m1*), is unsurprising given that newly synthesized Lrp2 must navigate the biosynthetic pathway to reach the apical membrane.

### Lrp2 Associates with Multiple Membrane Transporters through PDZ Binding Domains

We were surprised that many transporters were biotinylated by Lrp2, suggesting that these receptors are functionally linked to Lrp2. A functional interaction, in turn, may explain their requirement for Lrp2 for endocytosis. In the following examples, we provide evidence of a physical interaction with Lrp2 either directly or indirectly.

Slc9a3r1 (also called Nherf1, NHERF family PDZ scaffold protein 1) directly interacts with Lrp2 in vivo.^53^ SLC9A3R1 is involved in the trafficking and localization of various membrane proteins, including transporters and receptors, to the apical membrane of proximal tubule cells (Supplemental Table 3).^54^ Nherf1 also interacts with PDZK1, a scaffold protein biotinylated by Lrp2^APEX^.

Slc34a1 (also known as NPT2; FRTS2; SLC11; HCINF2; NAPI-3; NPTIIa; NPHLOP1; and SLC17A2) has a C-terminal PDZ-binding motif. It encodes a sodium-phosphate cotransporter critical for phosphate reabsorption in the kidney’s proximal tubules. Although there is no evidence of a direct physical protein–protein interaction between Slc34A1 and Lrp2, the downregulation of brush border membrane Slc34a1 by parathyroid hormone is dependent on Lrp2. In addition, both Slc34a1 and Lrp2 interact with Slc9a3r1 (Nherf1) and colocalize in the apical membrane by a post-transcriptional mechanism.^55,56^ In summary, all evidence suggests either a direct physical association or an interaction through a common partner.

Slc9a3, or NHE3, a sodium/hydrogen exchanger, has PDZ-binding activity and is localized in brush border membranes and vesicles. Upon stimulation by angiotensinogen, NHE3 redistributes to the tips of microvilli in the presence of Lrp2.^57^ Slc9a3 might interact with Lrp2 through Slc9a3r1, which is a PDZ scaffold protein, predicted to bind the PDZ-binding motif (S/T-X-Φ) in the cytoplasmic tails of Lrp2.^53^ Consistently, Lrp2 antibodies specifically precipitate NHE3 from rabbit renal cortical microsomes, but the interaction is not likely to be direct^58^ since the reciprocal immunoprecipitation using NHE3 antibodies did not pulldown Lrp2. In sum, Slc9a3 probably interacts with Lrp2 via an intermediate, since we were not able to demonstrate Lrp2-directed biotinylation of Slc9a3.

Clcn5, also known as Clc5, is a chloride channel that is mutated in human Dent’s disease, resulting in proteinuria. There appears to be a clear functional interaction between Clcn5 and Lrp2, as Lrp2 is reduced dramatically in the proximal tubules in Clcn5^–/–^ mice.^59^ It is possible that Slc9a3r2, another PDZ scaffold protein, mediates the physical interaction between Clcn5 and Lrp2.^60^ This possibility is highlighted by FDR = 0.066, implicating limited direct contact with Lrp2^APEX^.

### Lrp2-Mediated Endocytosis Initiates at the Microvilli

In addition to its distribution throughout the apical endocytic pathway, Lrp2 is present within the microvilli that constitute the apical brush border.^7,8^ Our experiments identified several brush border apical enzymes and ion transporters, including the amino acid transporter subunits B(0)­AT1 (*Slc6a19*) and rBAT (*Slc3a1*), sodium-phosphate transporter IIa (NaPiIIa; *Slc34a1*), sodium-glucose transporters Sglt1 and Sglt2 (*Slc5a1, Slc5a2*), and their interacting subunit Map17 (*Pdzk1ip1*) and the lactate transporter Smct2 (*Slc5a12*). The unconventional myosin motor, myosin VIIb (*Myo7b*), which localizes to microvilli tips and is responsible for maintaining microvillar structure, was also biotinylated.^61^ A close association between Lrp2 and some of these transporters may be facilitated by common binding to the PDZ domain-containing scaffolding protein NHERF3 (*Pdzk1*), since Lrp2, rBAT, NaPiIIa, and Map17 contain PDZ-binding motifs.

Lrp2 has long been known to localize at the microvilli (or brush border) in addition to the base of microvilli and intracellular vesicles, but its functional relevance in endocytosis is little understood. The enrichment analysis of Lrp2-dependent biotinylated proteins identifies the brush border and actin filaments as the cellular components and the molecular function of Lrp2-associated proteins. We propose that Lrp2 binds its ligand in the microvilli, traffics to the microvilli base by interaction with motor proteins, and then undergoes endocytosis. Although molecular details are needed, we provide some evidence of proteins associated with Lrp2 in microvilli.

### Additional Findings: Lrp2-Mediated Biotinylation of Transketolase and β-Keto-L-gulonate Decarboxylase (BKGD)

Our analysis identified two biotinylated proteins that showed the greatest fold-increase in biotinylation by APEX2 and should be the basis of additional studies. Transketolase (Tkt) and CK054 (BKGD) regulate pentose metabolism and the generation of substrates for glycolysis. Tkt generates NADPH, xylulose-5-phosphate, and ribose-5-phosphate. It is abundant in the kidney proximal tubule.^62^ CK054 was recently identified as the BKGD enzyme involved in nonphosphorylated pentose metabolism, producing NADH and the pentose sugar xylulose, then xylulose-5-phosphate.^63^ It is a novel finding that both pentose-shunt cytoplasmic enzymes are highly biotinylated by Lrp2^APEX2‑V5/+^. One could imagine that the ATP demand of endocytosis or the NADPH demand by excess Lrp2 cargo, which results in cell stress, could activate the two pentose pathways. While Lrp2 plays a metabolic role by the capture of vitamins and nutrients and their carriers, there is no known linkage between Lrp2 and the pentose-xylulose pathways. Our novel finding should be confirmed by analysis of protein–protein interactions and evaluation by cross-deletion experiments using endocytosis and cell ATP, NADH, and NADPH as readouts.

## Supplementary Material









## Data Availability

Raw proteomics data (mass spectra, peak files, and results files) associated with this manuscript have been deposited in MassIVE under the accession code MSV000098968 and ProteomeXchange number PXD067851. The LRP2^APEX2‑V5^ mouse has been deposited at Jackson Laboratories as JAX Strain name: Lrp2-APEX2; Jax Strain ID: 419746. The V5-APEX2-RAP bacterial expression construct is being deposited at AddGene with ID: 241358.

## Notes

S1, histology of kidneys from Lrp2+ and Lrp2^APEX2‑V5/+^mice; S2, identification of V5 peptide in protein extracts from Lrp2-APEX2-V5 mice; S3, pulldown of endogenous biotinylated proteins from APEX-lacking C57BL/6J mice and from Lrp2-APEX2 mice; S4, pathway analysis of proteins captured by Lrp2-APEX2 directed biotinylation; S5, endosomal markers; and S6, additional images of full-length immunoblots (PDF)
Table S1, Lrp2-mediated biotinylation of proteins (XLSX)
Table S2, endocytic machinery biotinylation by Lrp2-apex (XLSX)
Table S3, Lrp2-mediated biotinylation enrichment of proteins with PDZ domains (XLSX)
