# Supplementary material for: Proximity Labeling Reveals How Lrp2 Interacts with the Endocytic Machine
Source: J Proteome Res. 2026 Apr 20;25(5):2318–30. doi: 10.1021/acs.jproteome.5c01053 (PMC13140602; doi:10.1021/acs.jproteome.5c01053)
Supplement: Supplementary file 1 [file pr5c01053_si_002.pdf]

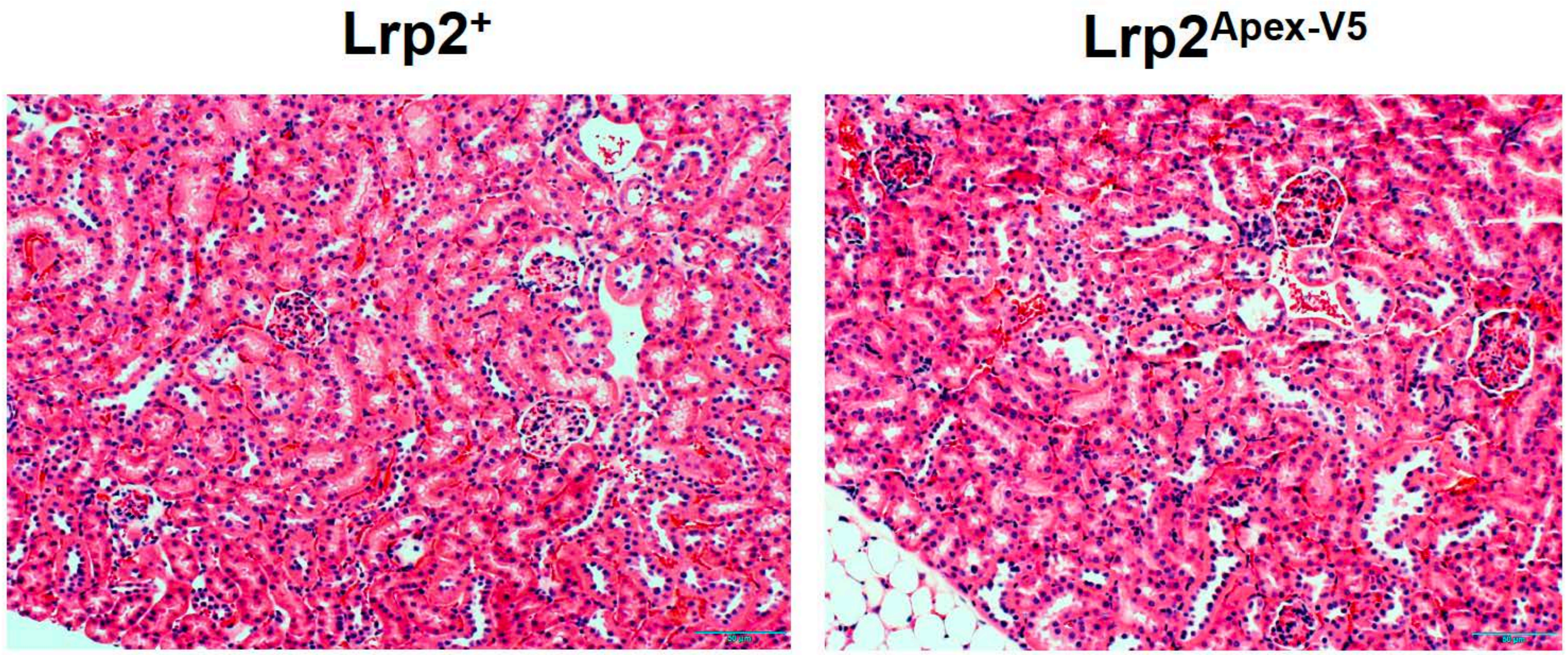

Supplemental Figure 1. Histology of kidneys from Lrp2+ and Lrp2APEX2-V5/+mice. No gross abnormalities were identified.

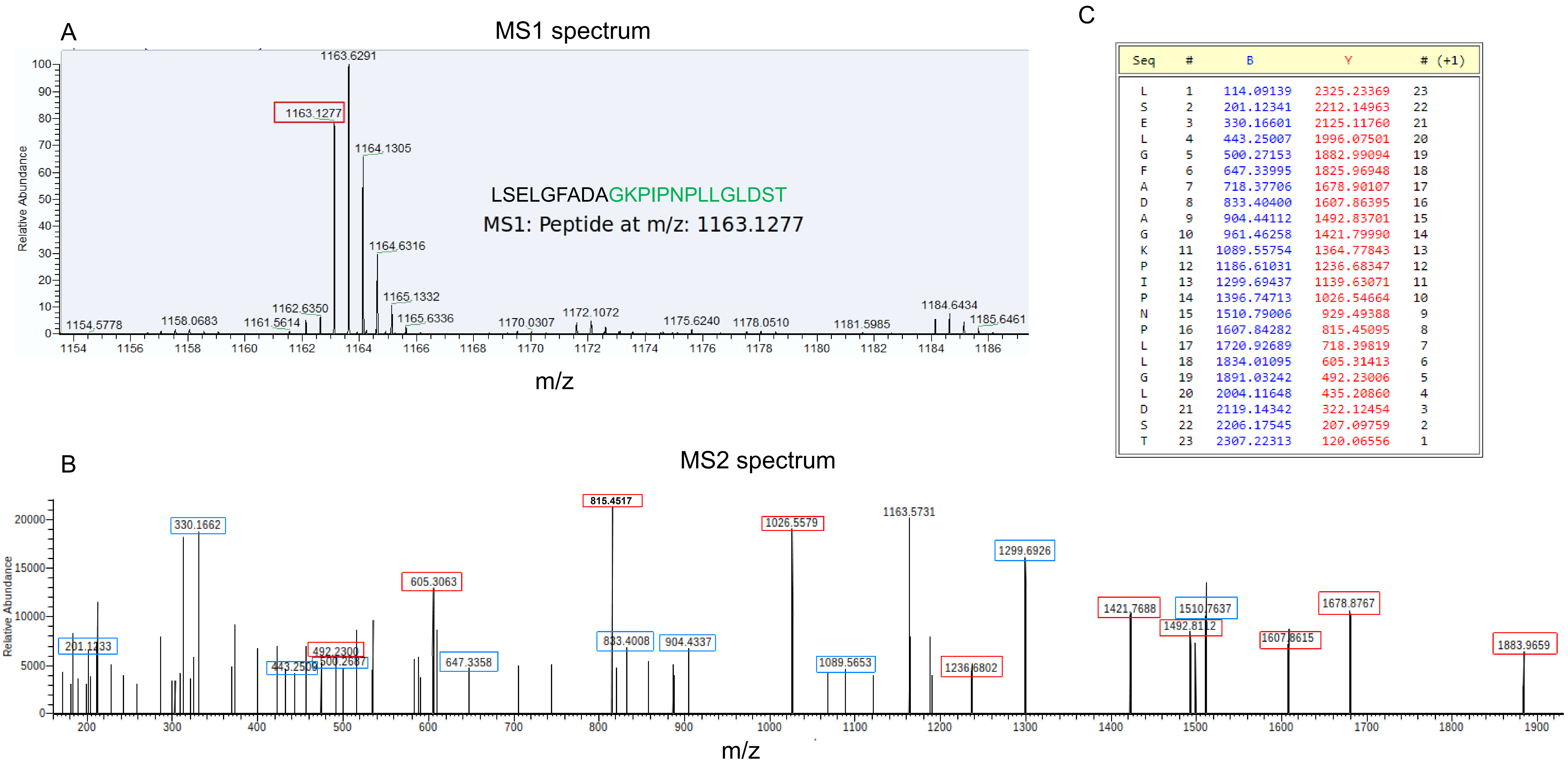

Seq	#	B	Y	# (+1)
L	1	114.09139	2325.23369	23
S	2	201.12341	2212.14963	22
E	3	330.16601	2125.11760	21
L	4	443.25007	1996.07501	20
G	5	500.27153	1882.99094	19
F	6	647.33995	1825.96948	18
A	7	718.37706	1678.90107	17
D	8	833.40400	1607.86395	16
A	9	904.44112	1492.83701	15
G	10	961.46258	1421.79990	14
K	11	1089.55754	1364.77843	13
P	12	1186.61031	1236.68347	12
I	13	1299.69437	1139.63071	11
P	14	1396.74713	1026.54664	10
N	15	1510.79006	929.49388	9
P	16	1607.84282	815.45095	8
L	17	1720.92689	718.39819	7
L	18	1834.01095	605.31413	6
G	19	1891.03242	492.23006	5
L	20	2004.11648	435.20860	4
D	21	2119.14342	322.12454	3
S	22	2206.17545	207.09759	2
T	23	2307.22313	120.06556	1

Supplemental Figure 2. Identification of V5 peptide in protein extracts from Lrp2-APEX2-V5 mice. The "V5" tryptic peptide LSELGFADAGKPIPNPLLGLDST was detected by DDA and by DIA acquisition methods as experimental mass in MS1. The peptide was confirmed by fragmentation spectra (MS/MS). (A) MS1 spectrum showing the precursor peptide ion at m/z 1163.1277. The amino acid sequence of the tryptic peptide is displayed with the V5 portion, colored in green. (B) MS2 fragmentation spectrum of the precursor peptide showing the b-ion series (boxed in blue) and y-ion series (boxed in red). The highest peaks (monoisotopic or +1 isotope) for each fragment ion are labeled in the MS2 spectrum. (C) Theoretical b-ion and y-ion ladder showing expected fragment masses.

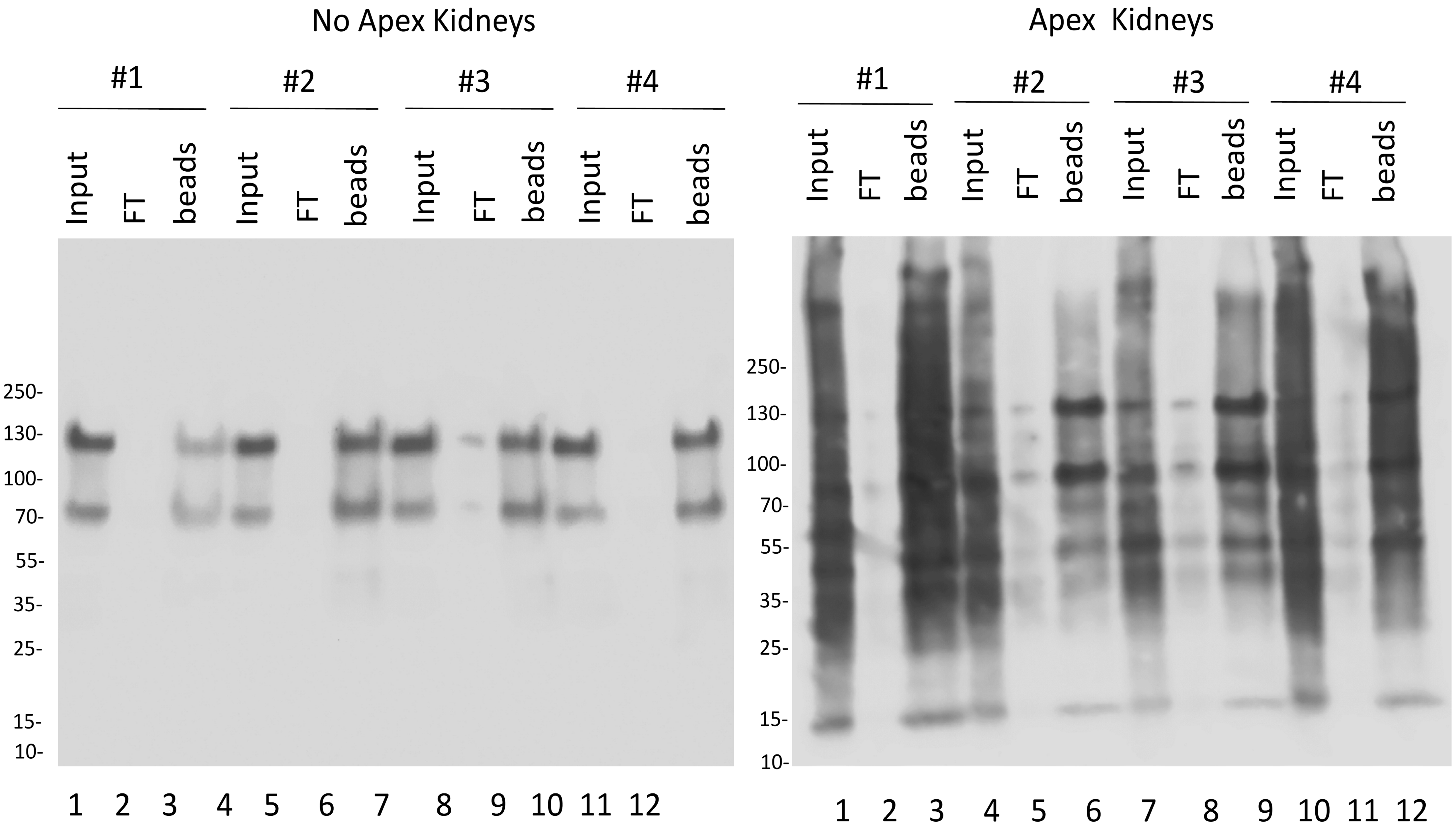

Supplemental Figure 3. A. Pulldown of endogenous biotinylated proteins from APEX-lacking C57BL/6J mice B. Pulldown of biotinylated proteins from Lrp2-APEX2 mice. Kidney lysates from male C57BL/6J mice or male Lrp2APEX2-V5/+ mice were biotinylated by sequential perfusion of biotin-phenol then H2O2. The biotinylated proteins were pulled down from lysates with streptavidin magnetic beads. Input (10.5 µg) and flowthrough (FT, 10.5 µg), and 10% of beads were analyzed by immunoblots using streptavidin-HRP (1:2000). Molecular weight markers (kDa) are on the left. Mice (n=4, each).

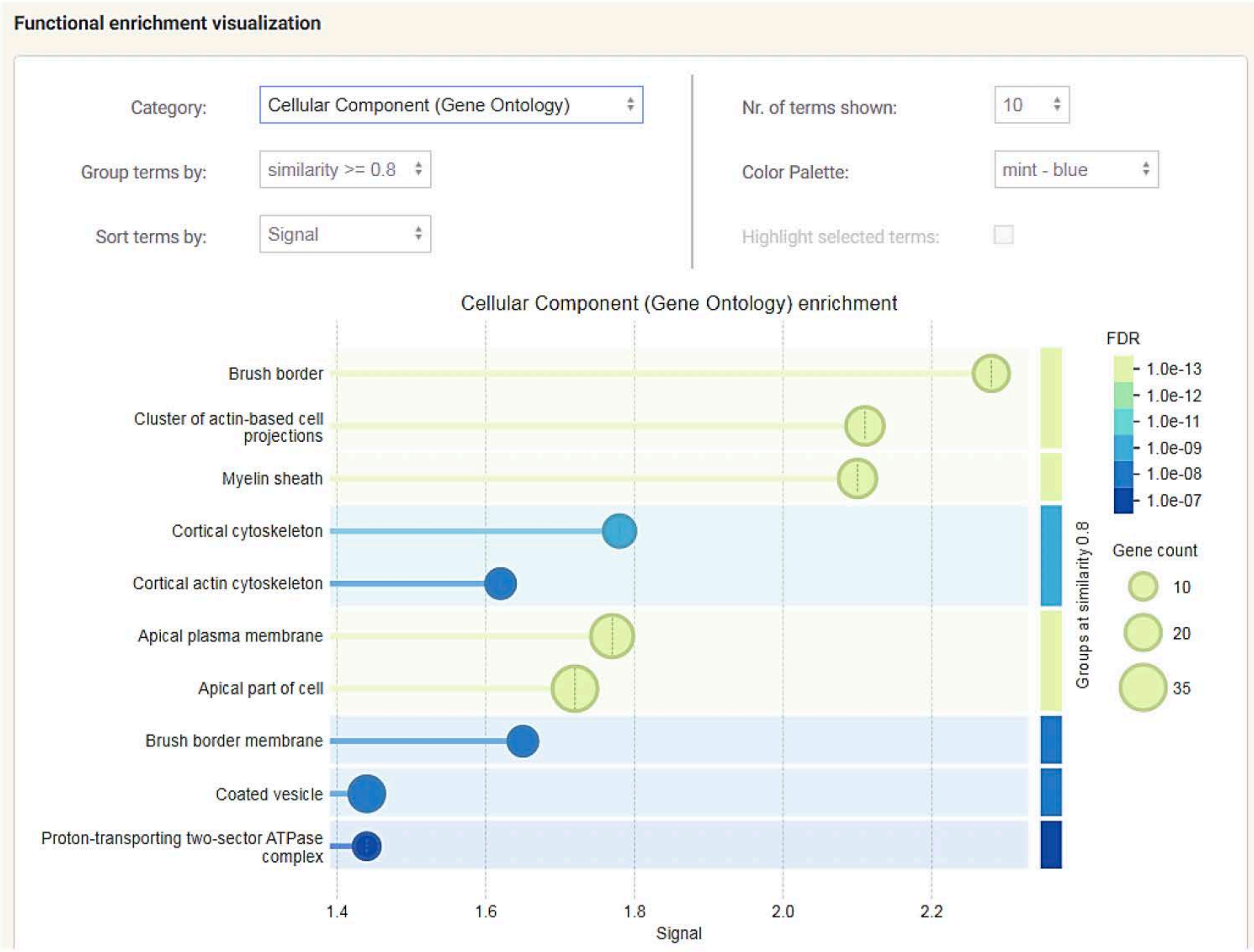

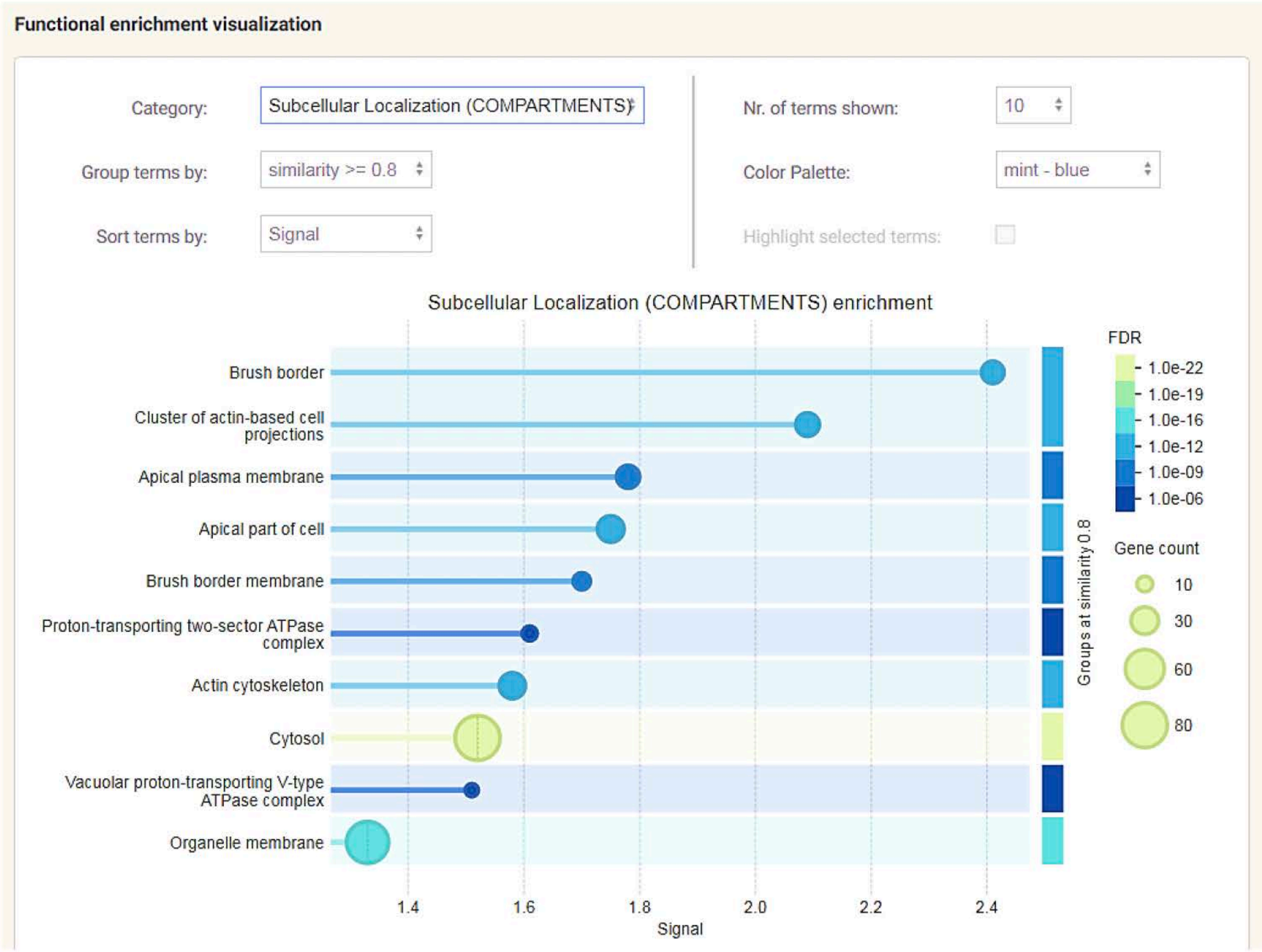

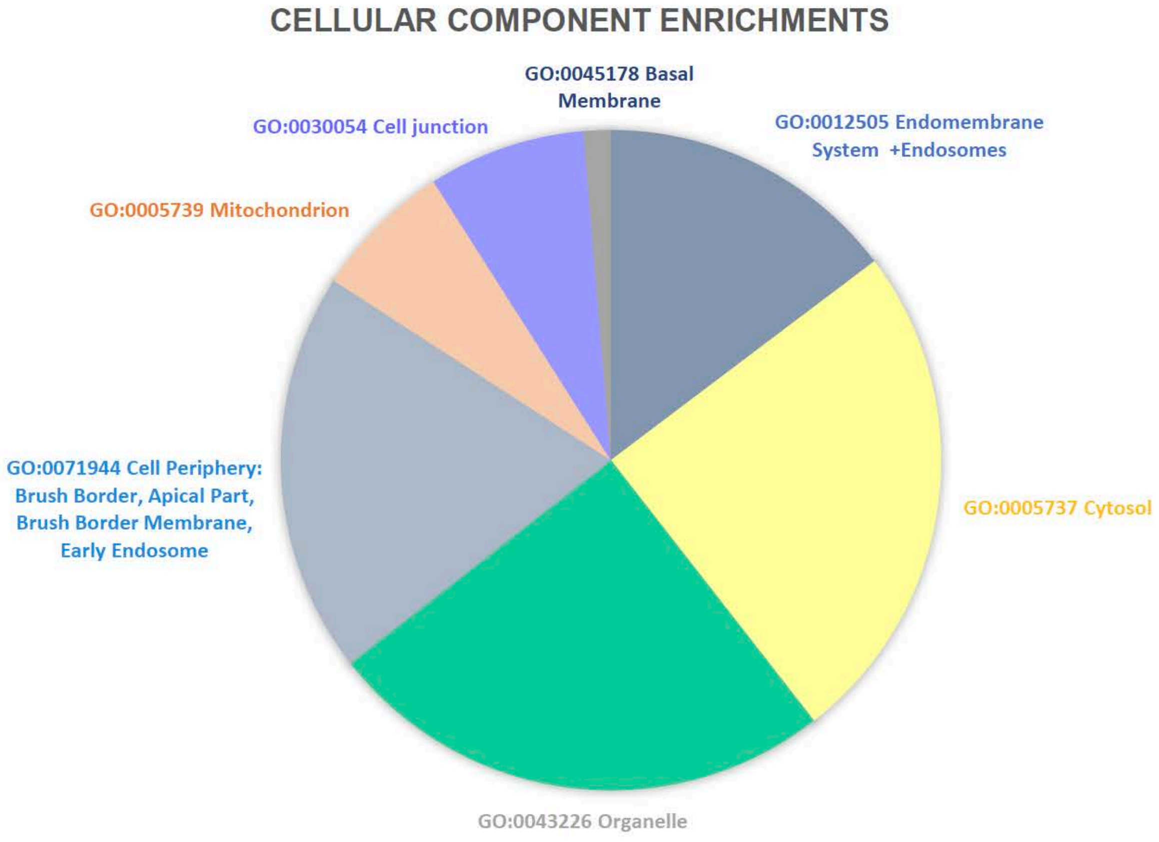

Gene Ontology #	Term description	Observed gene count in Lrp2-Apex
GO:0012505	Endomembrane System + Endosomes	233
GO:0005737	Cytosol	392
GO:0043226	Organelle	396
GO:0071944	Cell Periphery: Brush Border, Apical Part, Brush Border Membrane, Early Endosome	312
GO:0005739	Mitochondrion	108
GO:0030054	Cell junction	122
GO:0045178	Basal Membrane	21

Supplemental Figure 4. Pathway Analysis of Proteins Captured by Lrp2-APEX2 Directed Biotinylation. Proteins were ranked according to q-values. A selection of 248 biotinylated proteins with the most stringent q-values (q=0.01113-0.020) identified proteins associated with Brush border, Apical membranes, Coated vesicles and Myelin membranes, and the vacuolar ATPase, as well as cortical actin cytoskeleton, in line with the known locations of Lrp2 in the cell. Cellular component enrichments are also depicted as a pie chart according to observed gene count of biotinylated proteins, grouped by Gene Ontology designation as colored in Supplemental Table 2.

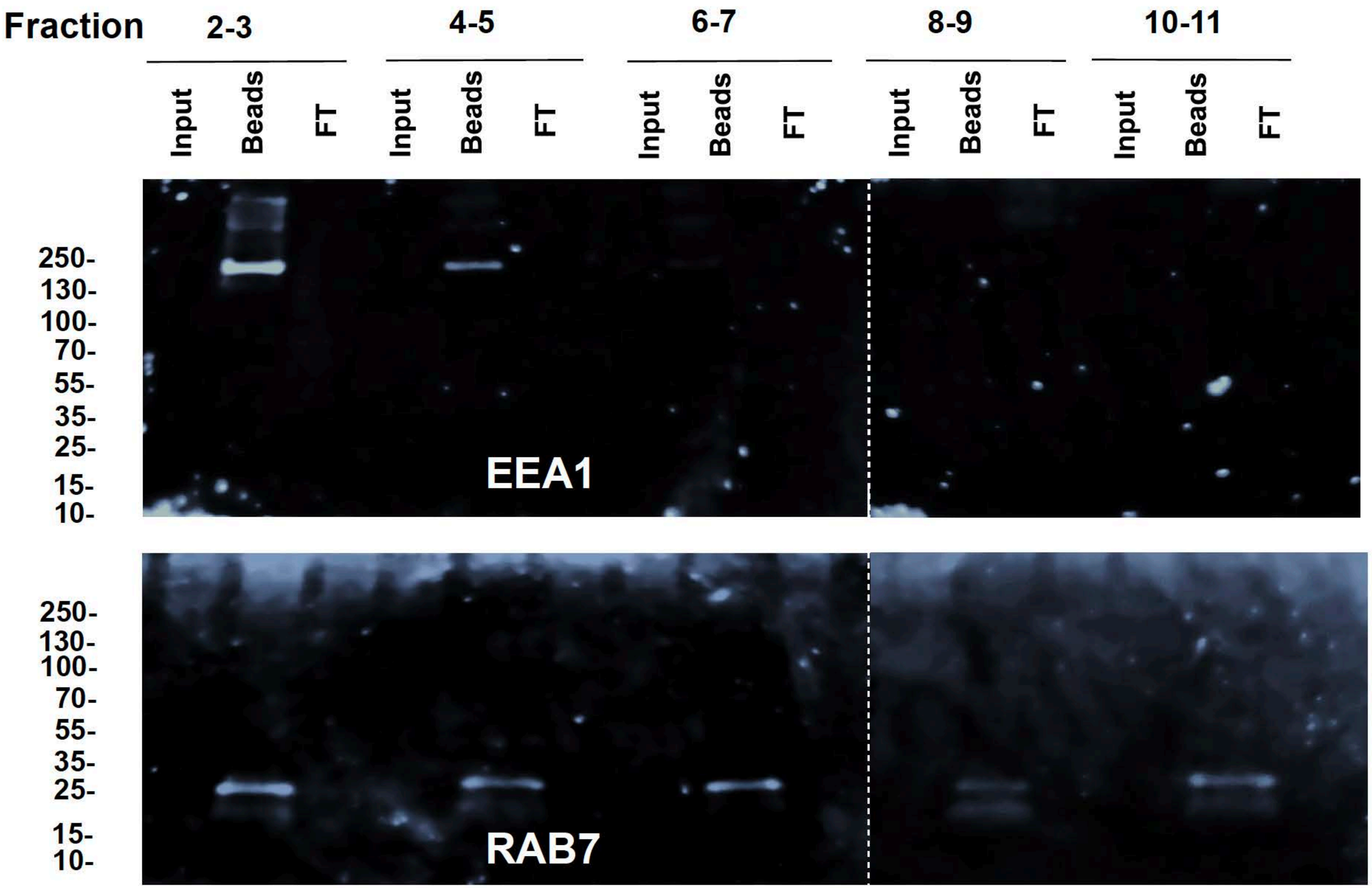

Supplemental Figure 5. Endosomal Markers. Lrp2APEX2-V5/+ endosomes were enriched by OptiPrep step gradients followed by anti-V5 nanobody bead pulldowns from the fractions. Eea1 and Rab7 are co-immunoprecipitated. Please refer to Figure 7.

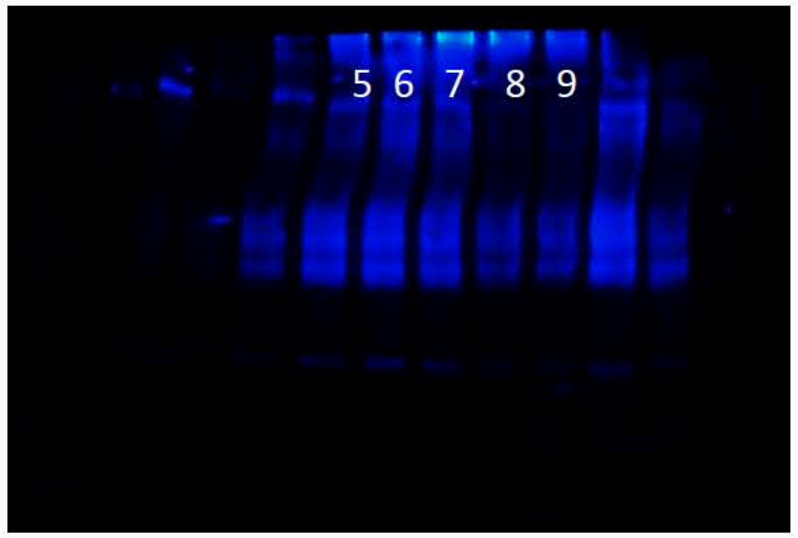

Full length immunoblot of Figure 7C

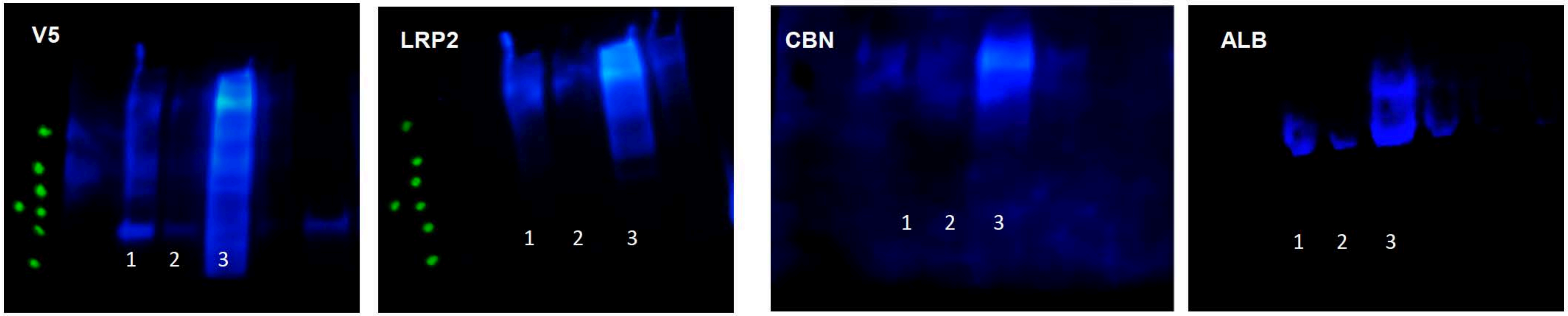

Full length immunoblot of Figure 7D

Supplemental Figure 6. Images of full length immunoblots from Figure 7.
